# Successful surgical treatment of descending aorta interruption in a 29-year-old woman with acute paraplegia and subarachnoid hemorrhage: a case report

**DOI:** 10.1186/s13019-015-0285-y

**Published:** 2015-06-06

**Authors:** Shutang Bai, Zhiheng Wang, Liang Zhang, Hongdu Fu, Huanwei Zhuang, Xianjun Cao, Liming Liang, Yanqi Yang

**Affiliations:** 1 Department of Cardiothoracic Surgery, Haikou Affiliated hospital of Xiangya Medical School, Central South University, Haikou, 570208 China; 2 US Department of Cardiothoracic Surgery, Linköping Heart Center, Linköping, Sweden

**Keywords:** Aortic interruption, Adult congenital heart disease

## Abstract

Interruption of the descending aorta is an extremely rare great vessel malformation. In this report, we describe a very unusual case of a 29-year-old female with a 13-year history of hypertension who was found to have an interruption of the descending aorta when she was hospitalized with a subarachnoid hemorrhage and symptoms of acute paraplegia. We successfully surgically corrected the defect using a Gore-Tex® graft to bypass the aortic interruption. The patient’s blood pressure postoperatively returned to normal, and the patient recovered completely from her paraplegia by the time of her 5-month follow-up visit.

## Background

Interrupted aortic arch is a rare, severe congenital heart defect defined as a complete loss of luminal and anatomic continuity between ascending and descending aorta [1], representing approximately 1 % of congenital heart disease [2], including 3 types as follows. Type A: aortic interruption occurs distal to the left subclavian artery. Type B: aortic interruption occurs between the left common carotid artery and the left subclavian artery. Type C: aortic interruption occurs between the innominate artery and the left common carotid artery [3]. One-stage repair including anastomosis for the aortic arch reconstruction and repair of all coexisting intracardiac defects is considered the treatment of choice [4]. Interruption of the descending aorta is a rare great vessel malformation, and a case including complications of acute paraplegia and subarachnoid hemorrhage is even more unusual. We herein describe one such case that we successfully treated using a Gore-Tex® graft to surgically bypass the aortic interruption.

## Case presentation

**Fig. 1:**
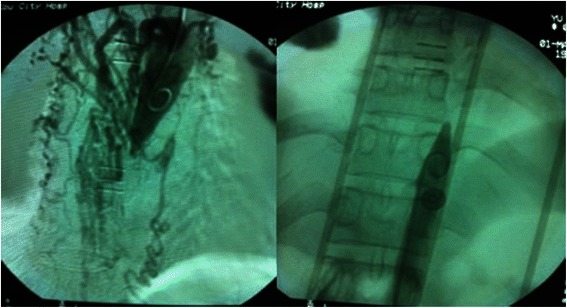
Aortography showing a bird’s beak appearance, total interruption of the descending aorta at level T_8–10_, and tortuous and dilated collateral vessels

A 29-year-old woman with a history of hypertension and previous subarachnoid hemorrhage 1 month ago was referred to our neurosurgical department because of symptoms including headache, vomiting, fecal and urinary incontinence, and paraplegia for 9 h on December 2, 2011. She had a history of hypertension for 13 years, and she had previously undergone two caesarean deliveries because of uncontrolled hypertension. On physical examination, she was conscious but nuchal rigidity was noted. Her heart rate was 62 beats/min with a regular rhythm and no heart murmurs. She had no tremor or cyanosis in her distal extremities. Her blood pressure was 165/88 mmHg in the upper extremities, and 94/59 mmHg in the lower extremities. She had no sensation to pain or light touch below the xiphoid process. Her muscle strength was normal at 5/5 in both upper extremities, but absent at 0/5 in both lower extremities. Kernig and Brudzinski signs were both positive. An electrocardiogram revealed sinus rhythm, an incomplete right bundle-branch block and LV hypertrophy. Non-contrast head CT demonstrated a subarachnoid hemorrhage and bilateral ventricle hematocele. Aortography showed a bird’s beak appearance and total interruption of the descending aorta at level T_8–10_, and the collateral vessels were tortuous and dilated (Fig. 1). The patient was diagnosed with descending aorta interruption, subarachnoid hemorrhage, paraplegia, and secondary hypertension. On admission, she had also been diagnosed with pneumonia.

**Fig. 2:**
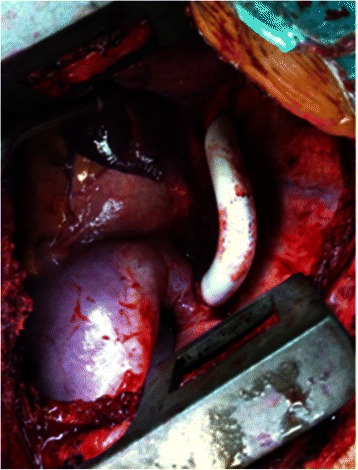
Descending aortic interruption bypassed with Gore-Tex® graft at surgery

She was transferred to our cardiothoracic surgery department. 15 days later, after treatment of her pneumonia, she underwent a successful operation without extracorporeal circulation. We made a posterolateral thoracotomy on the left chest. A 5 cm long, streak like coarctation located from 7 to 12 cm above the diaphragm of the descending aorta was found at the time of surgery. After opening the lesion, we found a complete interruption of the aorta rather than a coarctation. A thrombus which measured approximately 3.0 cm × 3.0 cm × 3.5 cm was also found proximal to the interruption site. We performed a bypass of this interruption using a Gore-Tex® graft that was 1.4 cm in diameter and 12 cm in length (Fig. 2). The postoperative pathological report indicated mixed thrombus with calcification.

**Fig. 3:**
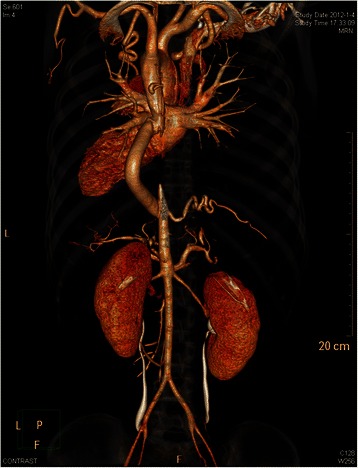
Postoperative chest computed tomography and three-dimensional reconstruction images demonstrating good graft patency

Postoperative blood pressure less than 24 h later improved to 120/70 mmHg in the upper extremities and 122/88 mmHg in the lower extremities. The patient began to recover sensory and motor function on postoperative day 3. Chest CT with three-dimensional reconstruction images on postoperative day 15 demonstrated good graft patency (Fig. 3). Three weeks after hospital discharge, her bowel and bladder function had recovered completely, her sensory function had recovered above the level of the knee, and her muscle strength was improved at 3/5 in the right calf and 2/5 in the left calf. Five months after discharge, the patient completely regained normal sensory function, and she had begun walking with only mild claudication symptoms.

### Discussion

Aortic interruption leads to elevated blood pressure proximal to the interruption, increased cardiac afterload, and ischemia of the limbs and abdominal organs distal to the interruption. Most patients are diagnosed in the neonatal period in critical condition due to congestive heart failure. Without timely treatment, the mortality rate is as great as 90 % at 12 months of life [5]. Thrombus was found proximal to the interruption during the operation, a result of blockage of blood flow at the existing total interruption of the descending aorta. Based on our intraoperative findings, including the site of interruption which was far from the aortic arch and agenesis of the interrupted descending aorta, we diagnosed this patient with a descending aorta interruption.

Interruption of the descending aorta should lead to increased blood pressure in the upper extremities and decreased blood pressure in the lower extremities. This patient had a longstanding history of hypertension in her upper extremities, and she had previously suffered a subarachnoid hemorrhage 1 month ago. However prior to this hospitalization, she had neither cardiac dysfunction nor decreased muscle strength in lower extremities. This lack of end-organ damage may be due to the fact that the interruption site is far from the aortic arch, at level of T_8–10_, where many thoracic collateral vessels would be able to bypass the interruption, decreasing cardiac afterload and improving perfusion of the lower limbs. This case should remind us that some patients, such as the one described in this case, may suffer severe consequences if we as physicians fail to investigate secondary causes of longstanding hypertension in the upper extremities. When faced with a young patient with unexplained hypertension, we should explore the possibility of aortic interruption. At minimum, evaluation should include measurement of the blood pressure in both upper and lower extremities.

Another important point for consideration we can draw from this case is the role of vascular surgery in the treatment of patients with acute paraplegia symptoms of vascular origin. The cervical spinal cord is supplied by the vertebral arteries, and the thoracic spinal cord is supplied by the intercostal arteries. The lower thoracic and lumbar spinal cord are supplied by the descending aorta and the internal branches of the iliac arteries. The anterior 2/3 of the spinal cord is supplied by the anterior spinal arteries, and the posterior 1/3 is supplied by posterior spinal arteries. Since blood supply is most vulnerable in transition zones being supplied by two different arterial sources, the lower thoracic spinal cord is a zone with a potential risk for paraplegia of vascular origin [6]. This is the same level that corresponds with our patient’s symptoms of paraplegia. Spinal cord ischemia can cause spinal cord infarction, and the resulting paraplegia is irreversible. Therefore, it is extremely unlikely that our patient could have recovered so well from paraplegia lasting 17 days if her symptoms were due to ischemia of the spinal cord. We suspect that her paraplegia symptoms were in actuality due to spinal cord edema caused by hypertension and elevated intracranial pressure. Perhaps this patient’s postoperative gradual improvement in spinal cord function was due to resolution of spinal cord edema as a result of her now normal blood pressure and decreasing intracranial pressure. Given this patient’s successful treatment, we can postulate that other patients with similar “vascular paraplegia” symptoms of short duration may benefit from surgical correction because they in fact have reversible “pseudo-paraplegia” symptoms of non-ischemic origin.

## Conclusions

When faced with a young patient with unexplained hypertension, we should explore the possibility of aortic coarctation or aortic interruption. Patients with hypertension of the upper extremities and paraplegia symptoms of short duration may in fact have “vascular paraplegia” symptoms that would benefit from surgical correction because they in fact have reversible “pseudo-paraplegia” symptoms of non-ischemic origin.

## Consent

Written informed consent was obtained from the patient for publication of this case report and any accompanying images. A copy of the written consent is available for review by the Editor-in-Chief of this journal.

## Abbreviations

LV: Left ventricular
CT: Computer tomography

## References

[1] Kleinrok A, Zaremba-Flis E, Smyk T (2010). Interrupted aortic arch in an adult female. Echocardiography.

[2] Kosucu P, Kosucu M, Dinc H, Korkmaz L (2006). Interrupted aortic arch in a adult: diagnosis with MSCT. Int J Cardiovasc Imaging.

[3] Mehrpooya M, Eskandari R, Salehi M, Shajirat Z, Golabchi A, Satarzadeh R, Zand-Parsa AF (2014). Undiagnosed interrupted aortic arch in a 59-year-old male patient with severe aortic valve stenosis: a case report and literature review. ARYA Atheroscler.

[4] Yasui H, Kado H, Yonenaga K, Kawasaki S, Shiokawa Y, Kouno H, Tominaga R, Kawachi Y, Tokunaga K (1993). Revised technique of cardiopulmonary bypass in one-stage repair of interrupted aortic arch complex. Ann Thorac Surg.

[5] Canova CR, Carrel T, Dubach P, Turina M, Reinhart WH (1995). Interrupted aortic arch: fortuitous diagnosis in a 72-year-old female patient with severe aortic insufficiency. Schweiz Med Wochenschr.

[6] Santillan A, Nacarino V, Greenberg E, Riina HA, Gobin YP, Patsalides A (2012). Vascular anatomy of the spinal cord. J Neurointerv Surg.

